# Functional Role of miR-499a-5p in the Development of Gastric Cancer

**DOI:** 10.5152/tjg.2024.24429

**Published:** 2025-01-01

**Authors:** Cuiling Lu, Chengwang Guo, Cuihua Wu, Liping Zhang, Xiaoling Liu, Shoucun Guo

**Affiliations:** 1 Digestive Endoscopy Center, Gansu Wuwei Tumour Hospital, Wuwei, China; 2 Department of Gastrosurgery, Gansu Wuwei Tumour Hospital, Wuwei, China

**Keywords:** miR-499a-5p, gastric cancer, functional, clinical significance

## Abstract

**Background/Aims:**

Gastric cancer (GC) is a highly prone malignant tumor, which has attracted wide attention. This study investigated the expression and clinical value of miR-499a-5p in GC.

**Materials and Methods:**

A total of 105 patients with GC were included in this study. Simultaneously, 55 patients with benign stomach disorders and 45 healthy subjects were enrolled as controls. Real-time quantitative polymerase chain reaction was used to determine the expression of miR-499a-5p. The receiver operating characteristic curve was used to assess the diagnostic value of miR-499a-5p in GC. Kaplan–Meier and logistic analyses were used to evaluate the association between miR-499a-5p and GC prognosis.

**Results:**

The levels of miR-499a-5p are markedly downregulated in GC and have a high diagnostic value. miR-499a-5p is closely linked to pathological features of GC. Overexpression of miR-499a-5p inhibits GC cell growth, migration, and invasion. Furthermore, miR-499a-5p is also related to GC and 5-year survival, and is a risk factor for GC death.

**Conclusion:**

The levels of miR-499a-5p were markedly downregulated in GC and related to GC pathological features. It has the potential to become a biomarker for the diagnosis of GC.

**Main Points**

- Serum miR-499a-5p is down-regulated in patients with GC.
- miR-499-a-5p is a potential biomarker for the diagnosis of GC.
- miR-499a-5p is closely linked to pathological characteristics of GC.
- miR-499a-5p linked to GC outcomes.

## Introduction

Gastric cancer (GC) is a common malignant tumor. In the last few years, although the occurrence and mortality of GC have decreased in Europe, the United States, and other developed countries, GC is still the third and fifth most common tumor in men and women, respectively, with about 1 million new cases of GC worldwide every year. The mortality of GC ranks second and fourth among the causes of malignant tumor death in all male and female patients, respectively. The number of patients who die from GC every year is as high as hundreds of thousands worldwide, causing great harm to human health.^1^ The incidence of GC ranks first among gastrointestinal tumors in China, seriously threatening people’s quality of life and survival.^2^ Despite significant progress in basic research and management of GC in recent years, overall treatment effects and prognosis remain poor.^3^ The main reason is that the specific cause and exact pathogenesis of GC are not fully understood, making early prevention and clinical treatment challenging.^4^ Therefore, studying tumor-related factors or phenotypic markers of GC is very helpful in supplementing and improving the occurrence, development, diagnosis, and treatment of GC.

The microRNA (miRNA) is an endogenous small RNA about 20-24 nucleotides in length, with various important regulatory functions in cells.^5^ As tumor markers, miRNAs have the advantage of being actively secreted by tumor cells. Their expression levels change constantly as tumor cells are produced and die, so each miRNA’s expression level reflects information about the body’s health or disease state.^5,6^ In recent years, research on miRNAs in GC and other tumors has made significant progress, enhancing the understanding of the molecular biology and genetic mechanisms of tumor development.^7^ These findings offer new insights into tumor pathogenesis and new targets for genetic diagnosis and treatment. Research shows that serum levels of miR-21 and miR-106 are markedly elevated in GC patients, and these levels markedly decrease after surgery.^8^ Therefore, serum miRNAs may be used as new diagnostic markers for patients with GC.

The onset and evolution of GC is a multifactorial, multigenetic, and multistage process, with invasion and metastasis being the main causes of death.^9,10^ Research has shown that miRNA-499 rs3746444 A/G polymorphism serves as a biomarker for predicting the recurrence of primary early GC after endoscopic submucosal dissection.^11^ In addition, it was demonstrated that GC cell-derived exosomes aggravated GC by transferring TTN-AS1 to GC cells via the miR-499a-5p/ZEB1/CDX2 axis.^12^ Earlier studies implicated miR-499a-5p in GC, but studies on its specific expression and role in GC are scarce. This study investigated the expression and clinical value of miR-499a-5p in GC.

## Materials and Methods

### Study Subjects and Data

In this study, 105 patients with GC diagnosed and treated in Gansu Wuwei Tumour Hospital were chosen to participate (GC group). The following criteria were used for inclusion: patients diagnosed by imaging examination, gastroscopy, and postoperative histopathological examination; patients with no history of GC who had not received surgery, radiotherapy, chemotherapy, or immunotherapy; patients with complete clinical data and clear TNM (Tumour Node Metastasis) staging; and patients and their families who could cooperate with the prognosis and follow-up of this study. In addition, the GC patients in this study received good psychological, nutritional, and drug care during and after treatment. Patients with other benign or malignant tumors and psychiatric disorders were excluded. Additionally, 55 patients with benign gastric diseases (including peptic ulcers, chronic gastritis, gastritis, gastric polyp) formed the control group (CON group), and 45 healthy individuals (healthy group) were enrolled as controls. Patients with a history of other malignant tumors and metabolic diseases were excluded. Fasting blood samples were taken from all subjects in the early morning.

The Gansu Wuwei Tumor Hospital ethics committee reviewed and approved this study (approval no: 2017-236) on March 13, 2017. The participants were informed about their rights and agreed to participate in the study. All subjects signed an informed consent form.

### RNA Extraction and Polymerase Chain Reaction

A Trizol kit (Invitrogen, USA) was used to extract total RNA. The quality of RNA was measured using NanoDrop 2000 (Thermo Fisher, USA). The NanoDrop 2000 measured the OD 260/OD 280 ratio between 1.8 and 2.1 and the OD 230/OD 260 ratio between 0.4 and 0.5. The samples then proceeded to the next stage of the experiment. Based on the extracted RNA template, miRNA cDNA was obtained using the Reverse Transcription Kit (Thermo Fisher). The expression of miR-499a-5p in the target samples was detected using real-time quantitative polymerase chain reaction. Confirmation was performed after the reaction was completed, and variable curves were drawn based on the obtained data. After three independent experiments, the data obtained were calculated and analyzed in combination with 2^−ΔΔCt^ expansion using U6 as the internal reference.

### Cell Culture and Processing

The GC cell lines MKN-45 and HGC-27 were obtained from Nanjing Key Gen Biotech Co., Ltd. The cells were routinely grown in PMI 1640 medium supplemented with 10% FBS in an incubator at 37°C in a humidified atmosphere with 5% CO_2_. Cells in the logarithmic growth phase were harvested after 48 hours, and the solution was changed under low light.

Overexpression of miRNAs was promoted by the miR-499a-5p mimic, while transfection with the mimic NC as a negative control did not affect the miR-499a-5p level. The synthetic miR-499a-5p mimic and mimic NC were from Guangzhou Ribo Bio Co., Ltd. in China. Either the miR-499a-5p mimic or the NC mimic was transfected into MKN-45 and HGC-27 cells. Transfection efficiency was measured after 24 hours of incubation.

### Cell Proliferation Assay

The Cell Counting Kit-8 (CCK-8) was carried out to evaluate cell viability. The CCK-8 reagent was added at 0, 24, 48, and 72 hours. The samples were incubated at 37°C for 1 hour, and the changes in absorbance at 450 nm were recorded.

### Transwell Cell Migration and Invasion Assays

All cell culture reagents and transwell chambers (pore size = 8 μm) (Millipore, Bedford, MA) were incubated at 37°C. Cells were grown to the logarithmic growth phase, digested, and washed once with PBS. Serum-free medium was used for the following experiments. The cells were counted, and the final concentration was made up to 2 × 10^5^/mL. Then 200 µL of each suspension was aspirated and added to the PET membrane of the transwell chamber. The membrane was uncoated for migration analysis and coated with diluted Matrigel for invasion analysis. About 600-800 µL of medium containing 10% FBS was added to the lower well and 100-150 µL of cell suspension to the upper well. This was then incubated for 24 hours. Five random fields were observed and counted under the microscope. The results were subjected to statistical analysis.

### Data Collection and Prognosis Follow-up

The clinicopathological data of the enrolled patients were collected. Their survival was monitored through return visits and telephone follow-ups. Follow-up began on the day of surgery and occurred every 3 months for 5 years.

### Statistical Analysis

SPSS 26.0 software (IBM SPSS Corp.; Armonk, NY, USA) and GraphPad 9.0 software (GraphPad Prism; Motulsky, San Diego, CA, USA)were used for statistical analysis. Measurement data were expressed as mean ± standard deviation. The independent samples* t*-test was used to compare 2 groups, while ANOVA compared multiple groups. The least significant difference test was used for pairwise comparisons after ANOVA. Real-time quantitative polymerase chain reaction was used to detect the expression level of miR-499a-5p. The receiver operating characteristic (ROC) curve was used to evaluate the diagnostic value of miR-499a-5p in GC. Logistic regression was used to analyze the risk factors for GC mortality. The relationship between miR-499a-5p expression and survival was assessed using the Kaplan–Meier method. Statistical significance was defined as *P* < .05.

## Results

### General Information

From the comparison of the general information of the 3 groups of subjects, it can be concluded that the GC group, CON group, and healthy group had no statistical significance in age, gender, smoking, drinking, triglycerides (TG), serum total cholesterol (TC), high-density lipoprotein (HDL), and low-density lipoprotein (LDL) (*P*>.05) (Table 1).

### Expression of miR-499a-5p

**Figure 1.**
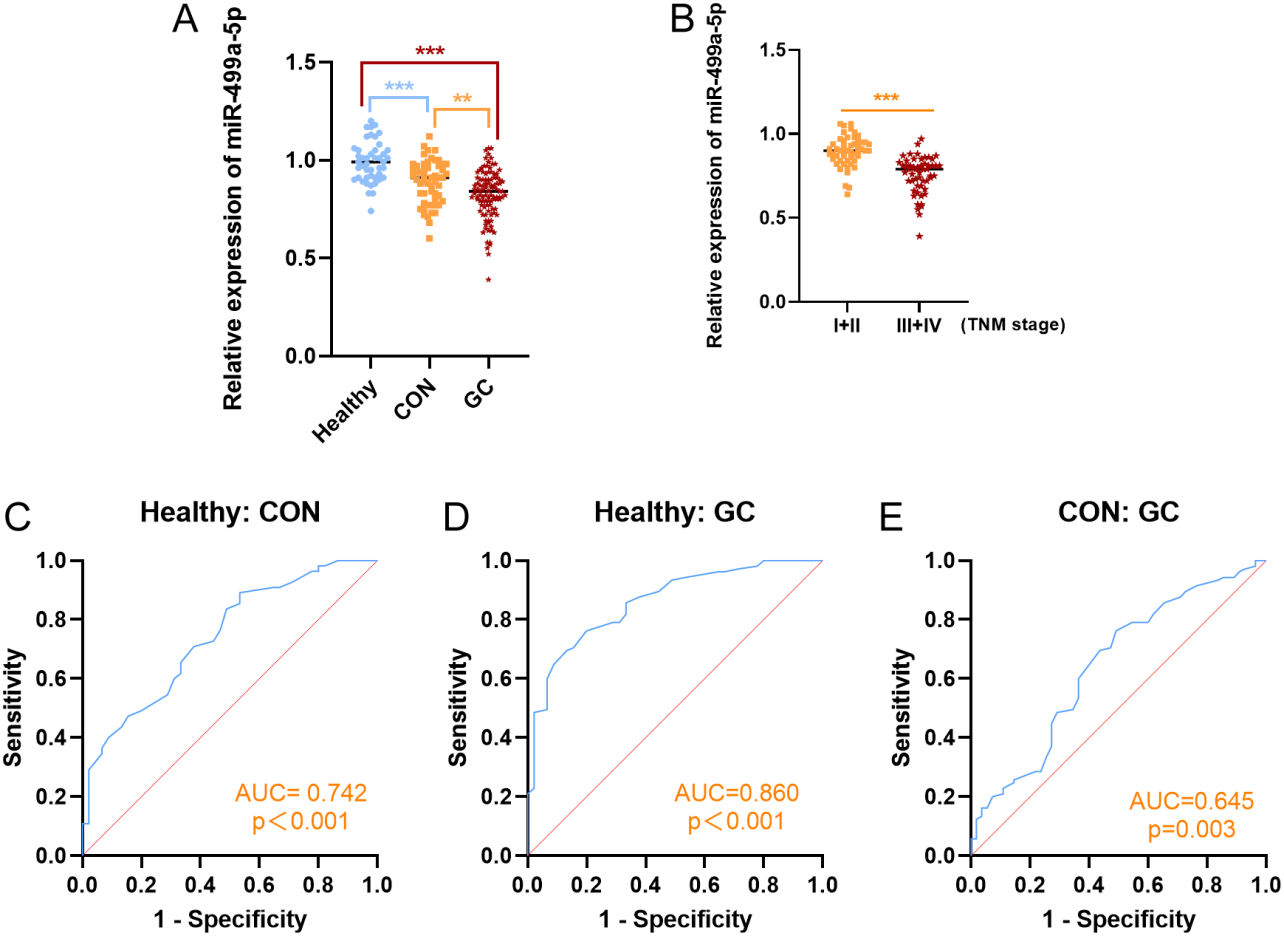
The expression and diagnostic value of miR-499a-5p. Compared with healthy controls, CON and GC serum miR-499a-5p were markedly reduced (*P* < .001) (A). The GC serum miR-499a-5p levels were lower than CON (*P* < .01). The serum miR-499a-5p was markedly lower in GC III + IV than in GC I + II (*P* < .001) (B). The AUC of serum miR-499a-5p in the diagnosis of CON and healthy individuals was 0.742 (95% CI = 0.647-0.838, *P* < .001), with a sensitivity of 70.9% and a specificity of 62.2% (C). The AUC of serum miR-499a-5p in the diagnosis of GC and healthy individuals was 0.860 (95% CI = 0.799-0.922, *P *< .001), with a sensitivity of 85.7% and a specificity of 66.7% (D). The AUC of serum miR-499a-5p in the diagnosis of GC and CON was 0.645 (95% CI = 0.554-0.737, *P *= .003), with a sensitivity of 64.8% and a specificity of 62.2% (E). ^**^P < .01, ^***^P < .001.

Figure 1A shows the levels of miR-499a-5p in the 3 subject groups. Compared with healthy controls, CON and GC serum miR-499a-5p were markedly reduced (*P* < .001). The levels of miR-499a-5p in the GC serum were lower than those in the CON (*P* < .01). In patients with GC, serum levels of miR-499a-5p decreased with the worsening of the severity of GC (*P* < .001) (Figure 1B).

### Diagnostic Value of miR-499a-5p

The ROC curve was used to analyze the accuracy of serum miR-499a-5p in diagnosing GC. The AUC of serum miR-499a-5p in the diagnosis of CON and healthy individuals was 0.742 (95% CI = 0.647-0.838, *P* < .001), with a sensitivity of 70.9% and a specificity of 62.2% (Figure 1C). For the diagnosis of GC and healthy individuals, the AUC was 0.860 (95% CI = 0.799-0.922, *P *< .001), with a sensitivity of 85.7% and a specificity of 66.7% (Figure 1D). In diagnosing GC versus CON, the AUC was 0.645 (95% CI = 0.554-0.737, *P *= .003), with a sensitivity of 64.8% and a specificity of 62.2% (Figure 1E).

### Relationship between Serum miR-499a-5p Level and Pathological Features of Gastric Cancer

Table 2 shows the results of the validation of miR-499a-5p levels with pathological features of GC. The level of miR-499a-5p was markedly related to depth of invasion, TNM stage and lymph node metastasis (*P*<.01). However, miR-499a-5p levels were not related to *Helicobacter pyroli *(HP) infection or tumor location (*P*>.05).

### Impact of miR-499a-5p on Gastric Cancer Cell Function

**Figure 2.**
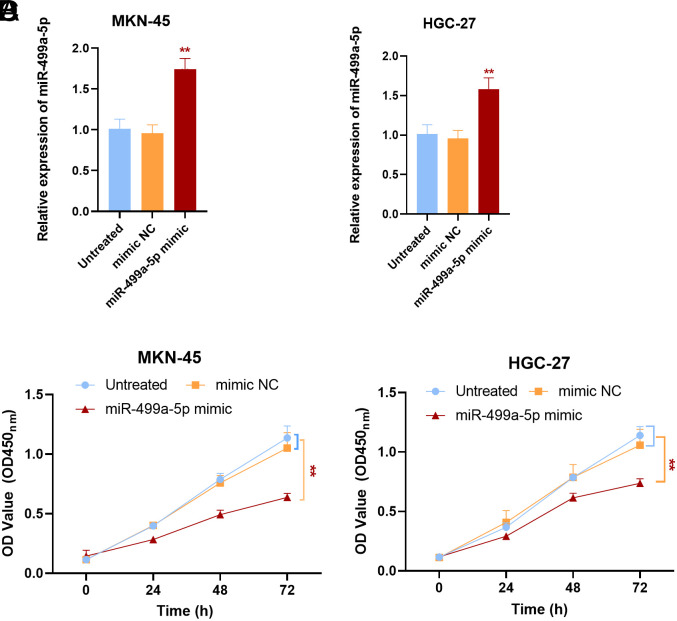
Cell proliferation experiment. Compared with the control groups, transfection of miR-499a-5p mimics markedly increased the expression level of miR-499a-5p in cells (A, B). Compared to the control groups, the upregulation of miR-499a-5p inhibited the growth of the cells (C, D). ^**^P < .01.

**Figure 3.**
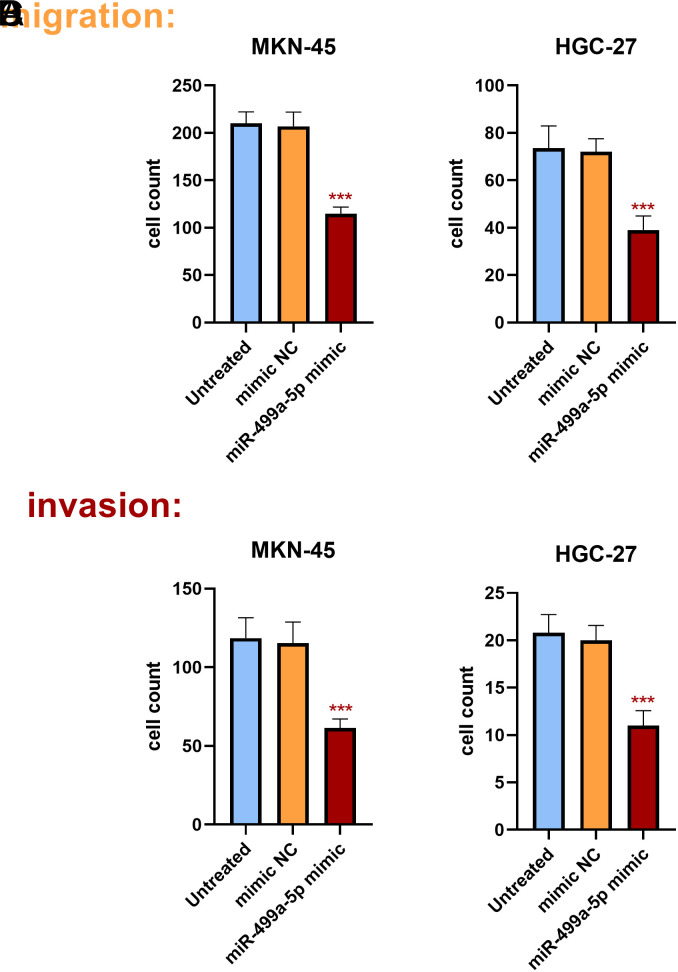
Transwell cell migration and invasion assays. Compared to the control groups, the upregulation of miR-499a-5p inhibited the migration of the cells (A, B). The upregulation of miR-499a-5p inhibited the invasion of the cells (C, D). ^***^P < .001.

The effect of miR-499a-5p on GC cell function was further investigated by cell experiments. In comparison to the control group, miR-499a-5p levels were markedly increased in cells transfected with miR-499a-5p mimics, indicating that the cells were effectively transfected (*P*<.01) (Figure 2A and B). Cell growth was inhibited after transfection with miR-499a-5p mimics in the CCK-8 assay. Upregulation of miR-499a-5p was confirmed to inhibit cell proliferation by analysis of the cell growth curve (*P*<.001) (Figure 2C and D). Transwell cell experiments showed that, compared with the control groups, the migration and invasion ability of GC cells were influenced by the upregulation of miR-499a-5p (* P*<.001) (Figure 3).

### Relation miR-499a to Gastric Cancer Prognosis

**Figure 4.**
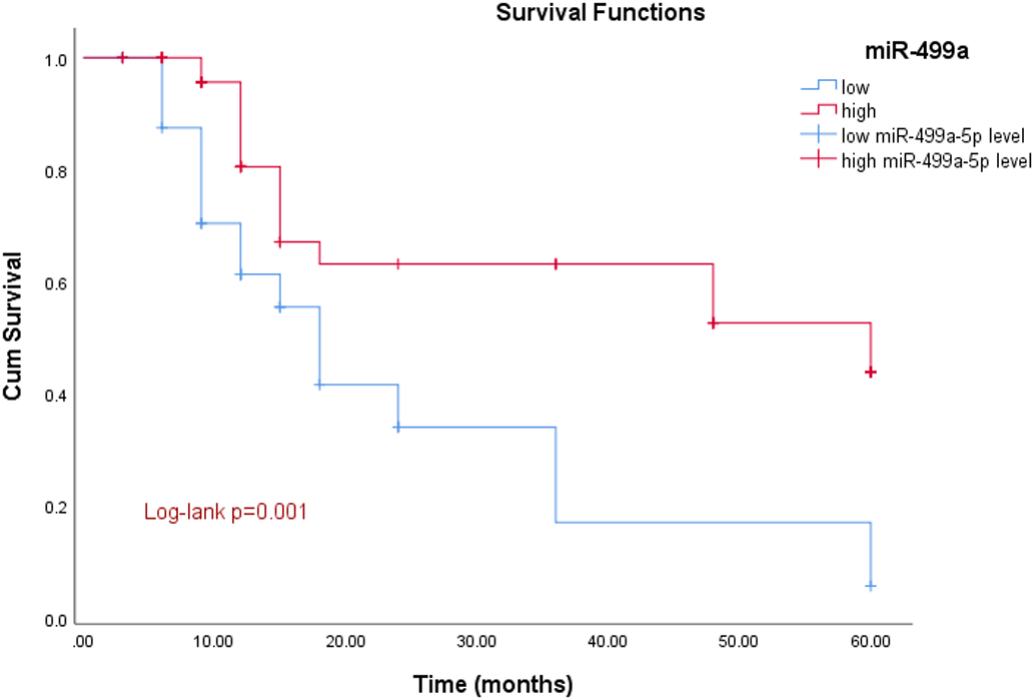
Kaplan–Meier survival curve. The survival time of patients with a low level of miR-499a-5p expression was markedly lower than that of patients with a high level of miR-499a-5p expression (log-rank *P *= .001).

Based on miR-499a-5p mean levels, 105 GC patients were divided into a miR-499a-5p high expression group and a miR-499a-5p low expression group. Among them, there were 51 cases in the high expression group and 54 cases in the low expression group. The Kaplan–Meier survival results showed that the survival rate of those expressing low levels of miR-499a-5p was considerably lower than that of those expressing high levels of miR-499a-5p (log-rank *P *= .001) (Figure 4).

### Analysis of Risk Factors for Gastric Cancer Mortality

With the survival rate of GC patients as the dependent variable, and HP, TNM stage, tumor location, and other factors as independent variables in binary logistic analysis, we discovered that miR-499a-5p was a risk factors for the 5-year survival of GC patients after surgery, as were HP infection, depth of invasion, TNM stage, and lymph node metastasis. Drinking may be a risk factor for GC survival rates, but there was no statistical significance (*P*>.05). However, age, gender, body mass index (BMI), TG, TC, HDL, LDL and tumor location were not risk factors for the 5-year survival in GC patients (*P*>.05) (Table 3).

## Discussion

Gastric cancer often has no obvious symptoms in its early stages. As a result, many patients are diagnosed at an advanced stage, leading to low survival rates.^13^ Early diagnosis is essential to improve patient survival rates.^14^ At the same time, good psychological, diet, and drug care are also very important to improve the survival rate of patients with GC.^15,16^ Currently, tumor markers, histopathology, and TNM staging are used to assess GC status and prognosis. However, these methods have disadvantages such as low sensitivity and specificity, lack of preoperative staging, and lack of universality.^17,18^ Therefore, finding new biomarkers for disease diagnosis and prognosis is essential.

The pathogenesis of GC is related to the abnormal expression of various genes.^19^ microRNAs have been demonstrated to be involved in initiating, developing, and progressing GC. Their clinical value in evaluating GC condition and prognosis is higher than traditional imaging staging and tumor markers.^20,21^ Certain miRNAs are highly associated with GC, and when GC cells appear in the body, their concentration in the blood becomes abnormal. Research shows that serum miR-551b-3p expression decreases in GC patients, suggesting it may be a diagnostic biomarker for GC.^22^ The research showed serum miR-144 was down-regulated in GC, indicating its potential clinical value as a prognostic biomarker.^23^ Similar to previous studies, we discovered that miR-499a-5p was markedly down-regulated in GC and had a high diagnostic value. This is an indication that miR-499a-5p could be a useful marker for diagnosing GC.

Studies show that decreased serum miR-647 expression is associated with poorer outcomes in GC.^24^ Chen et al^25^ discovered that reduced serum miR-204 levels were predictive of an unfavorable prognosis in GC. Feng et al^26^ discovered that the downregulation of serum miR-126 was linked to a poor prognosis of GC. To date, there is no study on the levels of miR-499a-5p in GC and its correlation with the disease and prognosis. This study demonstrated that GC with low levels of miR-499a-5p had a substantially lower 5-year survival rate. Additionally, we discovered that miR-499a-5p was a risk factor for death in GC. This indicates that miR-499a-5p is associated with the prognosis of GC.

In addition, miR-499a can regulate various target genes and pathways involved in tumor proliferation, invasion, and metastasis. Research shows that miR-499a inhibits prostate cancer proliferation by targeting UBE2V2.^27^ Wang et al^28^ found that miR-499a slows glioma cell proliferation by suppressing Notch1 and MAPK signaling. It has been shown that overexpressing miR-204-3p promotes apoptosis and inhibits the proliferation of GC cells through inhibition of MAPK and RIP1/MLK1 signaling pathways.^29^ Research has shown that miR-335-5p inhibits GC progression by targeting MAPK10.^30^ Our study showed that the proliferation, migration, and invasion ability of GC cells were influenced by the upregulation of miR-499a-5p. Additionally, miR-499a-5p is closely related to the pathological characteristics of GC. This is an indication that miR-499a-5p could be implicated in the occurrence and development of GC. However, its target genes or signaling pathways in GC have not been explored in depth in this study, which will be the direction of future research. In general, this study is subject to the following limitations. The sample in this study was relatively small and older, which may have biased the results. The applicability of the conclusions might be diminished because the research was based on a specific patient subset. In addition, miR-499a-5p is implicated in GC cell function, but the underlying mechanism remains unclear. Whether it can be used as a useful biomarker to help diagnose GC needs further study.

In conclusion, miR-499a-5p is markedly down-regulated in GC and can affect cell function, potentially serving as a biomarker for GC diagnosis. Additionally, future studies are needed to explore miR-499a-5p-based pathways and individualized treatment. The expansion of specific research directions, such as the joint study of miR-499a-5p and other biomarkers, will provide substantial value for the application of miR-499a-5p in GC.

## Figures and Tables

**Table 1.** Baseline Characteristics of Study Subjects

Variable	Healthy (n = 45)	CON (n = 55)	GC (n = 105)	*P*
Age	63.91 ± 10.50	64.55 ± 10.78	65.70 ± 10.09	.544
Gender (male/female)	28/17	27/28	61/44	.074
BMI (kg/m^2^)	21.87 ± 2.61	21.29 ± 2.24	20.92 ± 2.56	.101
Smoking (yes/no)	22/23	25/30	58/47	.367
Drinking (yes/no)	23/22	36/19	73/32	.096
TG (mmol/L)	1.58 ± 0.37	1.62 ± 0.36	1.71 ± 0.36	.098
TC (mmol/L)	3.72 ± 0.27	3.73 ± 0.30	3.78 ± 0.28	.305
HDL (mmol/L)	1.48 ± 0.16	1.44 ± 0.18	1.43 ± 0.18	.291
LDL (mmol/L)	2.71 ± 0.26	2.80 ± 0.29	2.83 ± 0.32	.154

BMI, body mass index; CON, patients with benign gastric diseases; GC, gastric cancer; HDL, high-density lipoprotein; healthy, healthy individuals; LDL, low-density lipoprotein; TC, serum total cholesterol; TG, triglycerides.

**Table 2.** Association between Serum miR-499a-5p Expression and Clinicopathological Characteristics in Patients with Gastric Cancer (n = 105)

Clinicopathological Features	Cases	MiR-499a-5p Level	95% CI	*R* ^2^	*P*
HP Infection				−0.013 to 0.085	0.020	.151
Yes	66	0.81 ± 0.13			
No	39	0.84 ± 0.11			
Tumor location			−0.076 to 0.019	0.014	.233
Body	52	0.84 ± 0.13			
Antrum	53	0.81 ± 0.12			
TNM stage			−0.195 to −0.114	0.356	<.001^***^
I + II	37	0.92 ± 0.07			
III + IV	68	0.77 ± 0.11			
Infiltration depth			−0.128 to −0.036	0.107	<.001^***^
T_1_-T_2_	43	0.87 ± 0.08			
T_3_-T_4_	62	0.79 ± 0.14			
Regional lymph node metastasis			0.030 to 0.125	0.092	.002^**^
Yes	67	0.79 ± 0.12			
No	38	0.87 ± 0.11			

HP, *Helicobacter pylori*.

**P < .01.

***P < .001.

**Table 3.** Logistics Regression Analysis of Risk Factors for Gastric Cancer Death

Variable	HR	95% CI for HR		*P*
		Lower	Upper	
miR-499a-5p	0.214	0.068	0.676	.009^**^
Gender	1.563	0.485	5.031	.454
Age	1.032	0.291	3.668	.961
BMI	1.108	0.343	3.577	.864
Smoking	1.432	0.456	4.491	.538
Drinking	0.342	0.103	1.137	.080
HP	0.245	0.072	0.842	.026^*^
HDL	1.695	0.464	6.195	.425
LDL	1.566	0.505	4.856	.437
TG	2.056	0.631	6.703	.232
TC	1.983	0.556	7.071	.292
TNM stage	0.218	0.066	0.715	.012^*^
Tumor location	0.404	0.107	1.520	.180
Infiltration depth	0.119	0.033	0.433	.001^**^
Regional lymph node metastasis	0.082	0.020	0.347	.001^**^

BMI, body mass index; HDL, high-density lipoprotein; HP, *Helicobacter pylori*; LDL, low-density lipoprotein; TC, serum total cholesterol; TG, triglycerides. *P < .05.

**P < .01.

## Data Availability

The data that support the findings of this study are available on request from the corresponding author.
